# Supplementary material for: DMRforPairs: identifying Differentially Methylated Regions between unique samples using array based methylation profiles
Source: BMC Bioinformatics. 2014 May 15;15:141. doi: 10.1186/1471-2105-15-141 (PMC4046028; doi:10.1186/1471-2105-15-141)
Supplement: Additional file 2 — DMRforPairs output for the comparison of A431-MCF7 and NA17018-NA17105. Please start from the HTML files in each folder. Available via the BMC Bioinformatics website. [file 1471-2105-15-141-S2.zip › 1394847754114233_MOESM2_ESM/A431_MCF7/figures/15246.pdf]

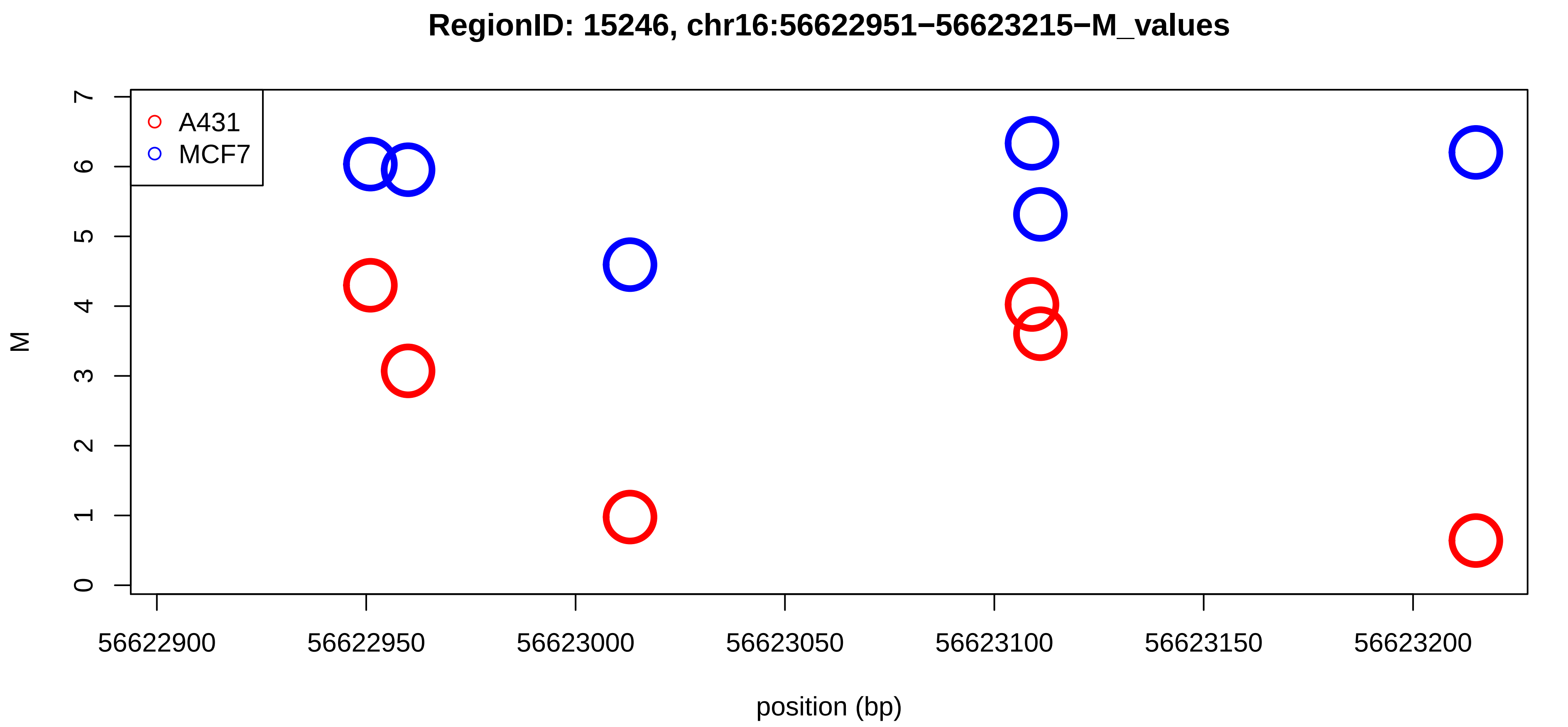
RegionID: 15246, chr16:56622951−56623215−M_values
A431
MCF7
M
0
1
2
3
4
5
6
7
56622900
56622950
56623000
56623050
56623100
56623150
56623200
position (bp)

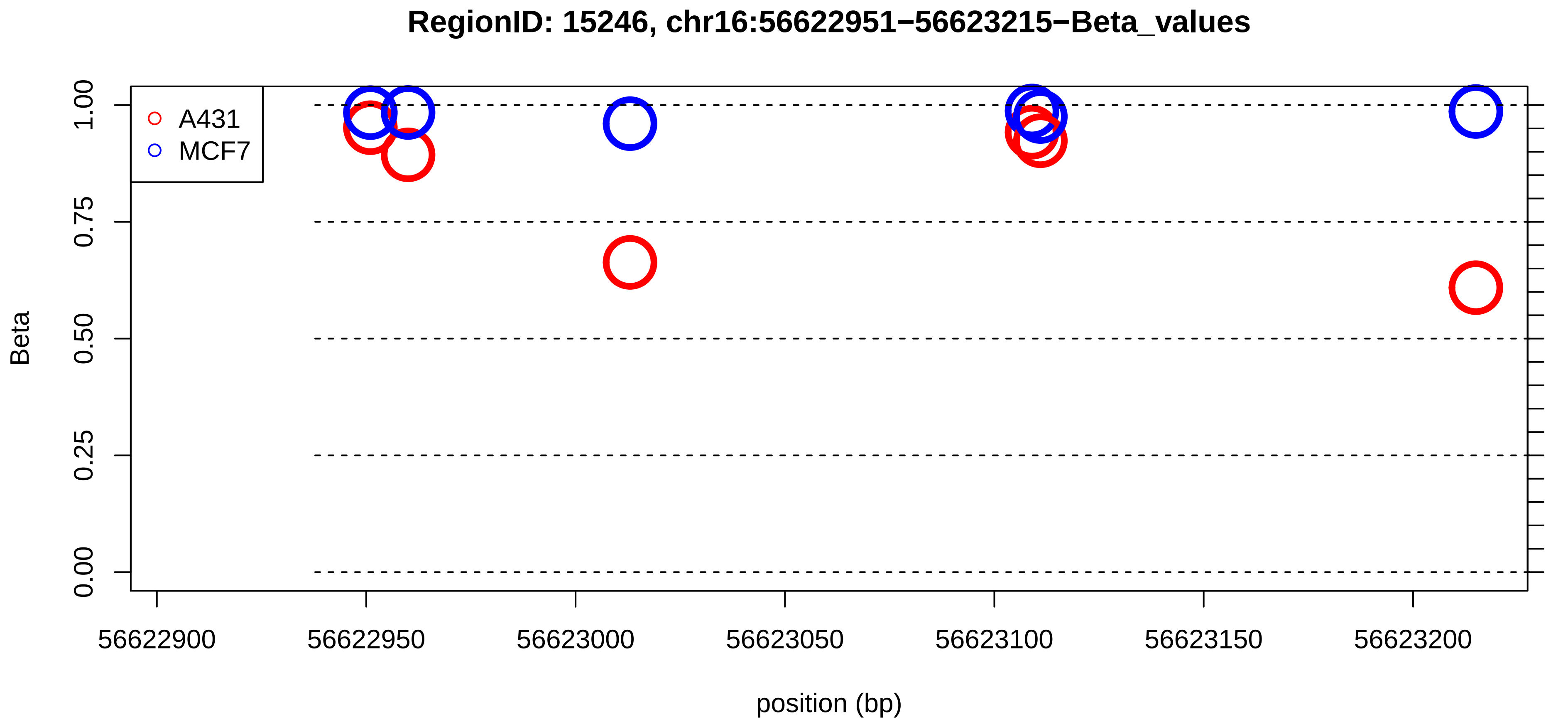
RegionID: 15246, chr16:56622951−56623215−Beta_values
A431
MCF7
Beta
0.00
0.25
0.50
0.75
1.00
56622900
56622950
56623000
56623050
56623100
56623150
56623200
position (bp)

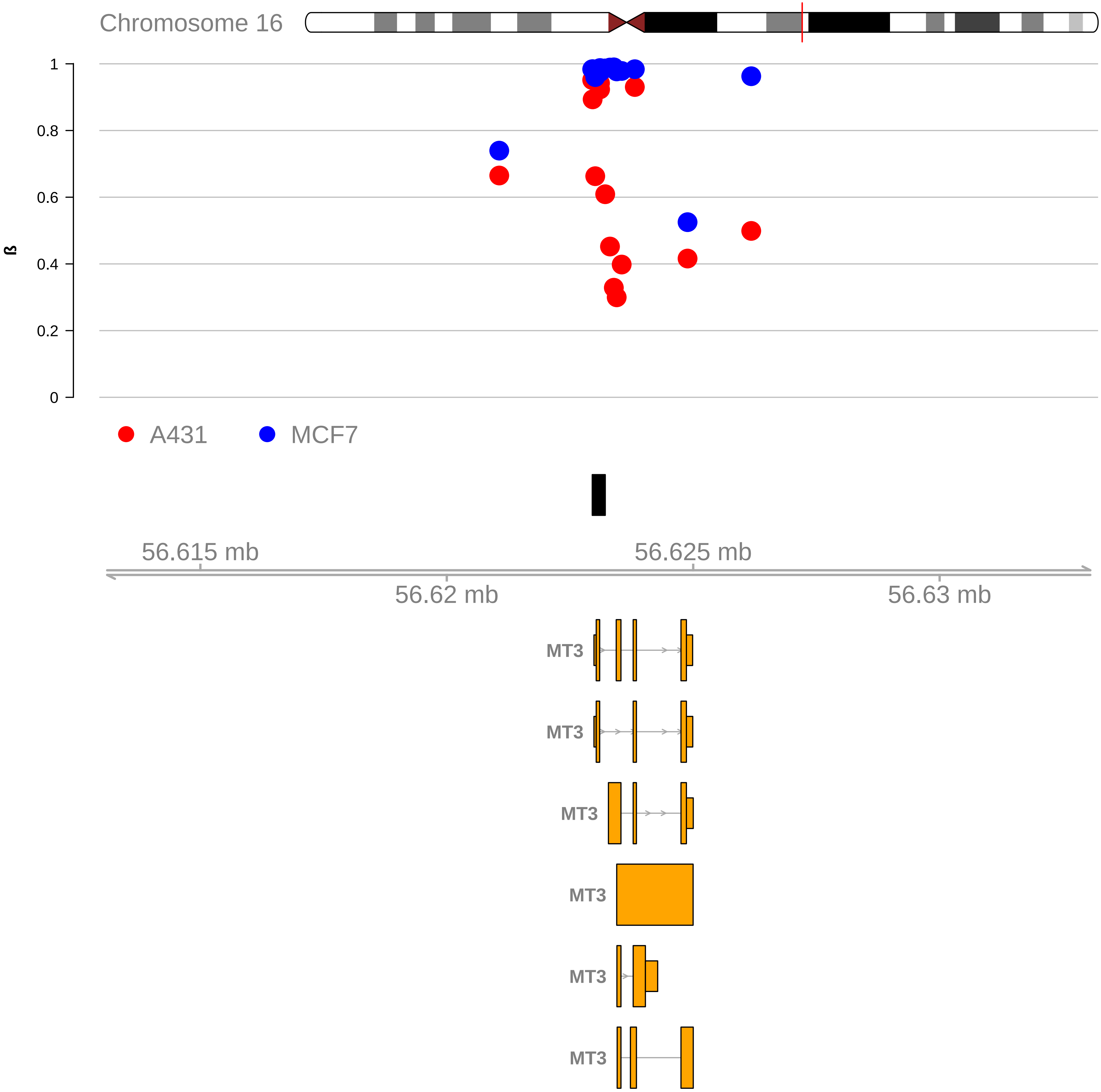

Chromosome 16
1
0.8
0.6
0.4
0.2
0
ß
A431
MCF7
56.615 mb
56.625 mb
56.62 mb
56.63 mb
MT3
MT3
MT3
MT3
MT3
MT3
